# Parkinson's disease and comorbid myasthenia gravis: a case report and literature review

**DOI:** 10.3389/fneur.2023.1303434

**Published:** 2024-01-08

**Authors:** Qihao Zhang, Erhe Xu, Hai-Feng Li, Piu Chan, Zhenzhen Zhao, Jinghong Ma

**Affiliations:** ^1^Department of Neurology, Xuanwu Hospital, Capital Medical University, Beijing, China; ^2^Department of Geriatrics Center, The Fourth People's Hospital of Shenyang, Shenyang, Liaoning, China

**Keywords:** parkinsonism, Parkinson's disease, myasthenia gravis, comorbidity, head drop

## Abstract

**Background:**

Parkinson's disease (PD) is the second most common neurodegenerative disease after Alzheimer's disease. Myasthenia gravis (MG) is a rare autoimmune disease caused by antibodies against the neuromuscular junction. PD and comorbid MG are rarely seen.

**Case presentation:**

Here we report on a patient who was diagnosed with PD and MG. A 74-year-old man had a 4-year history of bradykinesia and was diagnosed with PD. He subsequently developed incomplete palpebral ptosis, apparent dropped head, and shuffling of gait. The results of neostigmine tests were positive. Repetitive nerve stimulation (RNS) showed significant decremental responses at 3 and 5 Hz in the orbicularis oculi. The patient's anti-acetylcholine receptor (anti-AchR) antibody serum level was also elevated. Meanwhile, 9-[^18^F]fluoropropyl-(+)-dihydrotetrabenazine positron emission tomography–computed tomography (^18^F-AV133 PET-CT) scan revealed a significant decrease in uptake in the bilateral putamen. After addition of cholinesterase inhibitors, his symptoms of palpebral ptosis and head drop improved greatly and he showed a good response to levodopa.

**Conclusion:**

Although PD with MG is rare, we still need to notice the possibility that a PD patient may have comorbid MG. The underlying mechanism of PD and comorbid MG remains unknown, but an imbalance between the neurotransmitters dopamine and acetylcholine and the immune system are likely to play significant roles in the pathogenesis. In this article, we present our case and a literature review on the co-occurrence of PD and MG, reviewing their clinical features, and discuss the underlying pathogenic mechanism of this comorbidity.

## Introduction

Parkinson's disease (PD) is the second most common neurodegenerative disease after Alzheimer's disease, with an incidence of 17 cases per 100,000 person-years (1). Myasthenia gravis (MG) has an incidence of 8 to 10 cases per million person-years, making it even rarer (2). Thus, PD and comorbid MG is a rarely seen combination, with its prevalence grossly estimated at 3 in 6 million (3), representing a cumulative incidence of roughly 0.5 to 40 per billion (4). PD patients may present with fatigue, gait disturbances, dysphagia, dysarthria, and even limitations in eye movement, all of which can also be commonly seen in MG patients. The extremely low incidence and overlapping symptoms make it difficult for clinicians to diagnose this comorbidity. Since 1987, 57 cases of patients diagnosed with PD and comorbid MG have been reported. Coincidentally, we diagnosed a patient with PD and comorbid MG. We herein report our case of coexistence of PD and MG. We also summarize previously reported cases to enable further understanding of this comorbidity and discuss the underlying mechanism of pathogenesis.

## Case presentation

A 74-year-old man with a 4-year history of bradykinesia was diagnosed with PD and reported a poor response to levodopa. His medication was increased from a levodopa equivalent dose of 300 mg to 948 mg per day, with little improvement. One year before his admission, he developed incomplete palpebral ptosis of both eyes and gradually deteriorated in terms of ambulation. Three months before his admission, he also developed dysphagia, dysphonia, and head drop.

On neurological examination, the patient had normal cognition (the Mini-Mental State Examination score was 27/30, with 16 years of education). He presented with slight facial masking, incomplete palpebral ptosis, slurring of speech, dropped head, and bradykinesia. Other cranial nerves were normal. His muscle strength and tone of limbs were almost normal, but the strength of his neck extensor muscles was 2/5 and there was slight rigidity in his neck. He presented with no apparent tremor but took very small steps, as in freezing of gait. He had normal cerebellar examination, sensory faculties and reflexes. The Hoffmann signs and Babinski signs were negative bilaterally.

As the patient had responded poorly to levodopa (with <30% improvement) and there was no prominent tremor or rigidity, we gradually adjusted his anti-parkinsonism medication from levodopa-benserazide 250 mg (200 mg levodopa plus 50 mg benserazide) t.i.d., entacapone 0.2 g t.i.d., piribedil 50 mg t.i.d. to levodopa-benserazide 125 mg t.i.d. within 10 days. There was no visible deterioration in any of his symptoms after this reduction in drug dosage.

To determine why the patient had palpebral ptosis, we carried out several tests to exclude MG. Interestingly, administration of neostigmine significantly improved his head drop and palpebral ptosis as well as his gait (Supplementary Video 1). Repetitive nerve stimulation (RNS) showed significant decremental responses at 3 and 5 Hz in the orbicularis oculi. His anti-acetylcholine receptor antibody (anti-AchR antibody) serum level was >20 nmol/L (normal value: <0.45 nmol/L) and he was negative for anti-muscle specific tyrosine kinase (anti-MuSK) antibody.

**Figure 1:**
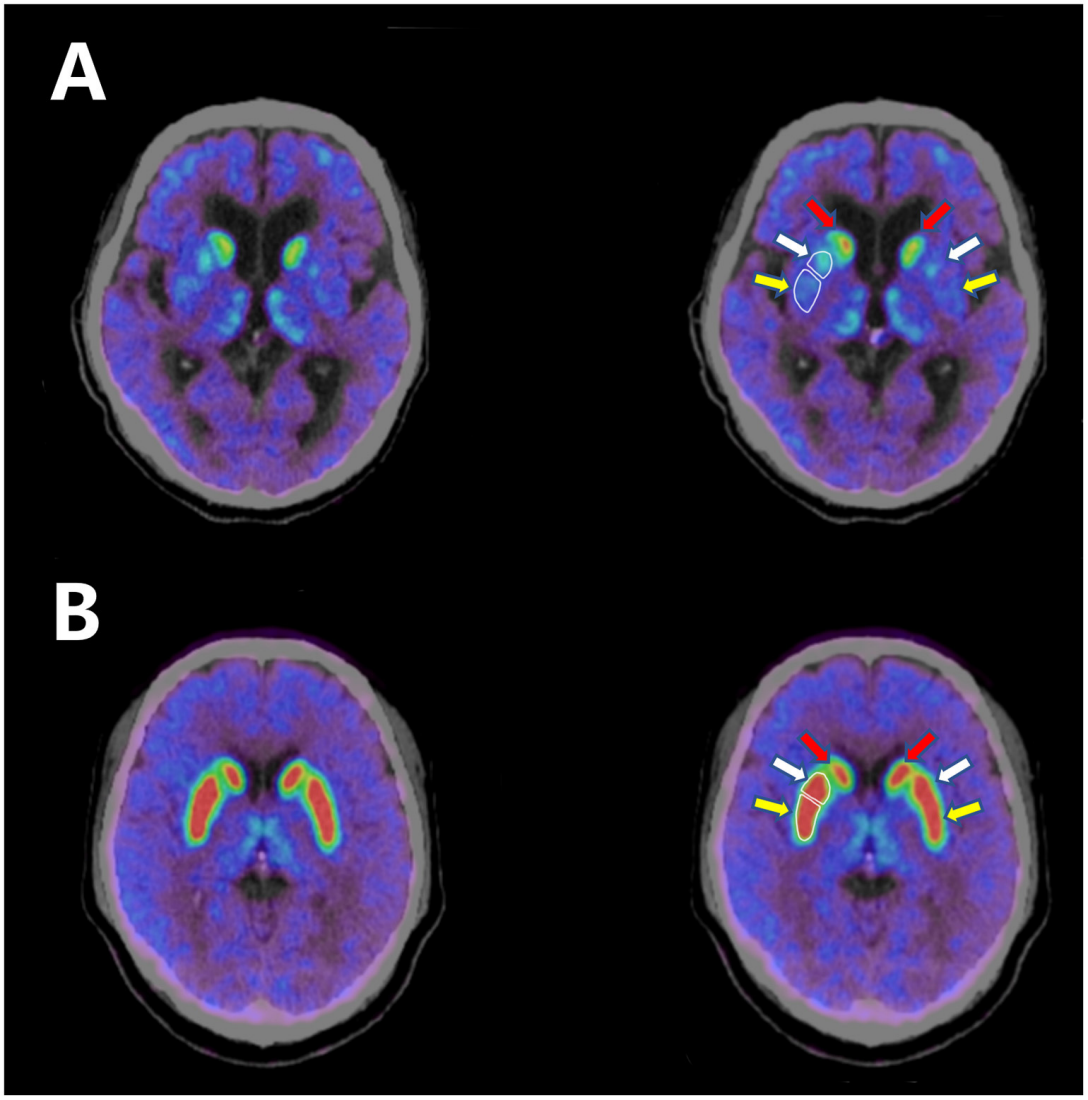
**(A)** ^18^F-AV133 PET-CT scan of the patient shows significant decrease in uptake in the bilateral putamen. **(B)** ^18^F-AV133 PET-CT scan of a healthy person shows symmetrical uptake in both the bilateral caudate nucleus and the putamen. White enclosed lines distinguish between the anterior putamen and the posterior putamen. Red arrow: caudate nucleus; white arrow: anterior putamen; yellow arrow: posterior putamen.

The patient was then treated with pyridostigmine 60 mg t.i.d. together with tacrolimus 3 mg q.d. One month later, his palpebral ptosis and head drop had improved greatly, but his parkinsonism symptoms had worsened and he experienced distinct “wearing off.” He presented with prominent rigidity in the neck and limbs when in the off state. We gradually increased levodopa-benserazide to 250 mg t.i.d., but his parkinsonism symptoms were still worse than the first time he visited our hospital. To verify the diagnosis of parkinsonism, we performed a 9-[^18^F]fluoropropyl-(+)-dihydrotetrabenazine positron emission tomography–computed tomography (^18^F-AV133 PET-CT) scan. The mean standardized uptake value ratios (SUVRs) for the left posterior putamen, right posterior putamen, left anterior putamen, and right anterior putamen were 2.14, 2.18, 2.15, and 2.45, respectively [the mean SUVRs for a healthy person are 5.12, 6.05, 5.36, and 5.36, respectively, and the reference diagnostic value distinguishing PD patients from healthy controls in the posterior dorsal putamen is 3.43 (5)], revealing a significant decrease in uptake in the bilateral putamen (Figure 1).

One and a half years after discharge from our hospital, the patient's palpebral ptosis and head drop had completely resolved, but he was experiencing bradykinesia, rigidity, postural instability, and freezing of gait. He received a levodopa equivalent dose of 1,066 mg, and the effect of each levodopa dosage could be maintained for 3 h. His bradykinesia, rigidity, and freezing of gait were improved, with a 32.2% change in the Movement Disorder Society-Sponsored Revision of the Unified Parkinson's Disease Rating Scale (MDS-UPDRS) III in a levodopa challenge test.

## Discussion and conclusion

PD has an incidence of 17 cases per 100,000 person-years (1), and MG has a much lower incidence of 8 to 10 cases per million person-years (2). Thus, PD and comorbid MG are rarely seen in clinical practice. We have described herein a patient diagnosed with PD and MG. In our case, positive outcomes of neostigmine tests, anti-AchR antibody levels, and RNS proved the diagnosis of MG. Bradykinesia, limb rigidity, and shuffling of gait were symptoms of parkinsonism. Decreased uptake in the putamen on ^18^F-AV133 PET-CT scan validated the impairment of the dopaminergic system. Motor fluctuation further confirmed the diagnosis of parkinsonism. As there were no absolute exclusion criteria or red flags, the patient was ultimately diagnosed with PD combined with MG.

In clinical practice, patients with PD may develop fatigue, gait disturbances, dysphagia, dysarthria, and even limitations of eye movement, which are also symptoms of MG; because of the rarity of PD comorbid with MG, it is difficult to consider a diagnosis of MG in PD patients with such symptoms. As fatigue is a frequent non-motor symptom of PD, the weakness presented by our patient initially did not remind us of the pathological fatigue occurring in MG.

We searched PubMed using “Parkinson's disease” and “myasthenia gravis” as key words and found 57 cases of this comorbidity from 1987 to Nov 20^th^, 2023. A retrospective observational study of 12 cases from a single center was also retrieved (6). Most of the cases were of male patients. Most patients were diagnosed with PD prior to the diagnosis of MG, and most had generalized MG. We summarize their demographic and clinical characteristics in detail in the Supplementary material.

To obtain more detailed information on PD and comorbid MG, we reviewed previous cases and identified a number of similarities and differences among the previously reported cases, presented in Table 1. Out of all these previous cases, nearly 80% (45/57) were of male patients. Their age at onset of their first disease ranged from 49 to 90 years old. In more than 70% of the cases (41/57), PD preceded MG, and this was also the case in our patient. Our patient had generalized MG and exhibited dropped head. More than 75% of the cases (44/57) had generalized MG, and approximately 30% (17/57) exhibited dropped head. More specifically, seven patients presented with dropped head as their first symptom of MG and six presented with dropped head as their sole symptom. In former cases, the diagnosis of PD generally depended on the clinical manifestations, neurological examinations, and good responses to levodopa, while in our case, we performed an ^18^F-AV133 PET-CT scan and confirmed impairment of the dopaminergic system. Regarding the diagnosis of MG, in addition to the typical clinical features of the patients, this was based on an increased anti-AchR antibody serum level in approximately 67.3% of the cases (35/52). The 19 patients who underwent an edrophonium test or neostigmine test all had positive outcomes. In 24 cases, RNS and/or single-fiber electromyography (SFEMG) were also performed, and the positive rate was 62.5% (15/24). Most patients responded well to cholinesterase inhibitors (pyridostigmine and/or neostigmine), or to immunosuppressive therapy such as steroids and azathioprine. In <20% of the cases, intravenous immunoglobulin or even plasma exchange was needed to control symptoms of MG.

**Table 1:** Similarities and differences among previously reported cases.

**Total number of cases**	**57**	**Ab of myasthenia gravis tested**	**52**
**Average age at onset of PD (years old)**	68.9	Anti-AchR Ab positive (%)	35 (67.3)
**Average age at onset of MG (years old)**	69.6	Anti-MuSK Ab positive (%)	1 (1.9)
**PD preceding MG (%)**	41 (71.9)	Seronegative MG (%)	16 (30.8)
**Patients exhibiting dropped head (%)**	17 (29.8)	**RNS/SFEMG conducted**	24
Dropped head as first symptom of MG (%)	7 (12.3)	RNS and/or SFEMG positive (%)	16 (66.7)
Dropped head as sole symptom of MG (%)	6 (10.5)	**Neostigmine/edrophonium test, Ab and EMG positive**	7^b^
**Administration of L-dopa**	28^a^	**Good response to MG treatment (%)**	39^c^
Good response to L-dopa (%)	28 (100)	Good response to single anticholinesterase drugs (%)	16 (41.0)
**Neostigmine test/edrophonium test conducted**	19	Good response to anticholinesterase drugs,	17 (43.6)
		glucocorticoids, and/or immunosuppressants (%)	
Neostigmine test/edrophonium test positive (%)	19 (100)	Requiring other treatment^d^ (%)	6 (15.4)

^a^Only 28 cases mentioned administration of L-dopa. ^b^The number of cases with neostigmine/edrophonium test, Ab, and EMG positive. ^c^Treatment of MG was mentioned in relation to only 41 cases, and 39 cases showed improvement or remained stable. ^d^Other treatments included intravenous immunoglobulin and/or plasma exchange.

Notably, the dropped head was a prominent sign in our patient, and dropped head in PD can be explained by either dystonia of the flexor neck muscles or weakness of the extensor neck muscles (7); however, the incidence of dropped head in PD appears to be <5 or 6% (7). Additionally, prominent or isolated neck extensor weakness is also distinctly less common in MG (8), with an estimated occurrence rate of only 10% (9). This occurrence rate rises to 23% for patients with an age of onset of MG of over 60 years (9). However, review of the previous cases of PD and comorbid MG showed that approximately one-third of the patients exhibited dropped head. Even so, we considered the differential diagnosis carefully. Disproportionate antecollis in parkinsonism, which presents as a form of head drop, is considered relatively rare in PD, while it is thought to occur more frequently in multiple system atrophy (MSA) (10). As a result, we had considered the diagnosis of our patient to be MSA at first, given his mild dysuria over the past 6 months, the relatively early presence of dysphagia, dysphonia, and head drop in his disease course (with a total duration of 4 years of parkinsonism), and his poor response to levodopa at the time of admission. Palpebral ptosis also looks similar to eyelid apraxia, which can also occur in MSA. Although the patient did not have prominent autonomic symptoms, he had no orthostatic hypotension, and the residual bladder volume was 15.6 mL. His finger-to-nose test and heel–knee–shin test were normal. All the evidence above made the diagnosis of MSA unlikely. Furthermore, head drop is common in amyotrophic lateral sclerosis (ALS), but the patient had no upper or lower motor neuron signs. Specifically, after his medication against MG was started, the patient exhibited significant improvement in his dropped head. Interestingly, in previous cases presenting with dropped head, we found that the symptom was almost always improved after treatment against MG, and cholinesterase inhibitors and/or immunosuppressive medications were used in most cases. In one case (11), the authors clearly pointed out that the patient's head drop responded weakly to levodopa, and in only one case (12), head drop was ameliorated by a custom head brace rather than pyridostigmine or intravenous immunoglobulin.

Although the comorbidity of PD and MG is unusual and seems like a coincidence, it is likely that there is an underlying mechanism. An imbalance between the neurotransmitters dopamine and acetylcholine (Ach) might be one of the reasons for the development of MG symptoms. In the second and the third case reported by Odajiu et al. (13), we noticed that both patients' anti-AchR antibody and RNS results were negative, with only the neostigmine tests positive. These patients' MG symptoms were improved by cholinesterase inhibitors. Therefore, we could hypothesize that the MG symptoms were induced by the relatively low level of Ach as treatment of PD raised the level of dopamine. In addition, it has been reported in previous cases that the anti-parkinsonism drug trihexyphenidyl (THP) induced or worsened the MG symptoms (3, 14). The first case reported by Ueno et al. (14) was that of a PD patient who developed MG 1 week after treatment with THP. Although the patient was positive for anti-AchR antibodies and thus it appeared to be unreasonable to suspect iatrogenic MG here, we could not ignore the fact that the severity of MG presented by the patient was closely related to the serum level of THP, which seemed to interfere with neuromuscular transmission through competition with acetylcholine receptor sites (14). In the case reported in 1993 (3), pyridostigmine worsened the patient's PD symptoms. However, after the MG symptoms of the patient were brought under control via steroids and pyridostigmine was withdrawn, the dosage of medications used to control PD symptoms was also decreased. In another case, pyridostigmine also appeared to cause an imbalance between dopamine and Ach by increasing Ach levels, thus inducing or worsening parkinsonism (15). Imbalanced interactions between the cholinergic and dopaminergic systems are observed in the pathological condition of PD (16). Thus, imbalanced interactions of the two systems may be one of the pathogenic mechanisms of PD with comorbid MG.

Another hypothesis with respect to the pathogenesis is autoimmunity. MG is an autoimmune disease and autoimmunity is also involved in the mechanism of PD. In six of the cases of PD and MG, the authors revealed that their patient had another autoimmune disease [Graves' disease (17), rheumatoid arthritis (18), psoriasis (6), polymyalgia rheumatica (6), or thyroiditis (6)]. It has been found that the risk of PD increases with some autoimmune disorders (19), and patients with an autoimmune disease generally have a 33% excess risk of PD (20). Accumulating evidence has suggested the significant role of the immune system in PD pathogenesis, either through inflammation or via an autoimmune response (21). α-synuclein (α-syn), the pathological hallmark of PD, is a potential target of autoimmune attack (22). Accumulation of misfolded α-syn is a trigger for PD and is involved in the pathology of neurodegeneration through innate and adaptive immunity (23, 24). Neuromelanin, another autoantigen released from dead dopaminergic neurons, is phagocytized by dendritic cells and subsequently induces the activation of microglia, leading to the autoimmune aggravation of PD (22). In addition to autoantibodies directed at α-syn and neuromelanin, antibodies directed at GM1 ganglioside have also been identified in PD patients. These antigens are associated with PD pathogenesis (25). Chen et al. (26) have reported that when rats receive plasma antibodies from PD patients, they undergo a marked loss of dopaminergic neurons. In contrast, rats that are injected with antibodies from healthy controls undergo much less neuronal damage, suggesting that PD patients may generate autoantibodies that attack their own dopaminergic cells (26). Sulzer et al. have found that T cells from patients with PD recognize α-syn peptides, thus mistakenly attacking brain cells and leading to the progression of PD (27). They have also confirmed this result through analysis of blood from PD patients, which they found to contain large numbers of T cells able to respond to α-syn (28). In the 57 cases of PD and comorbid MG, 70% of the patients developed MG after the onset of PD. It is likely that MG is secondary to the autoimmune mechanism of PD in these cases. Of course, more evidence is needed to prove the relationship between autoimmunity and PD with MG.

In conclusion, although PD with MG is rare, we still need to keep in mind that it is possible for a PD patient to have comorbid MG. Most of the patients reported have developed MG after the onset of PD. Meanwhile, head drop and palpebral ptosis are not common phenomena in PD, and thus comorbid MG should be suspected in such situations. The imbalance between the neurotransmitters dopamine and Ach may be the reason underlying comorbidity of PD and MG, and autoimmune mechanisms may also be involved in the pathogenesis. As a curable disease, MG should not be neglected when it occurs against the backdrop of PD.

## Data availability statement

The original contributions presented in the study are included in the article/Supplementary material, further inquiries can be directed to the corresponding author.

## Ethics statement

Ethical review and approval was not required for the study on human participants in accordance with the local legislation and institutional requirements. Written informed consent from the patient/participant or patient's/participant's legal guardian/next of kin was not required to participate in this study in accordance with the national legislation and the institutional requirements. Written informed consent was obtained from the individual for the publication of any potentially identifiable images or data included in this article.

## Author contributions

QZ: Writing—original draft. EX: Conceptualization, Data curation, Writing—review & editing. H-FL: Conceptualization, Data curation, Writing—review & editing. PC: Conceptualization, Data curation, Writing—review & editing. ZZ: Conceptualization, Data curation, Writing—review & editing. JM: Writing—review & editing.

## Supplementary material

Supplementary Video 1 Two neostigmine tests. This video shows two neostigmine tests. In the first neostigmine test, the patient had apparent head drop and incomplete palpebral ptosis at baseline. Twenty min after administration of neostigmine, both the head drop and the ptosis improved. After starting treatment with pyridostigmine for 10 days, we performed another neostigmine test; the footage shows that the patient took very small steps, as in freezing of gait, at baseline. Twenty min after administration of neostigmine, his gait improved.

Supplementary Table 1 Demographics and clinical features of previously reported cases^*^. This table shows demographics and clinical features of previously reported cases of PD with MG in details, including their sex, age, age at onset of PD/MG, PD clinical features, MG clinical features, MG diagnosis, PD treatment and MG treatment.
