# Supplementary material for: Parkinson's disease and comorbid myasthenia gravis: a case report and literature review
Source: Front Neurol. 2024 Jan 8;14:1303434. doi: 10.3389/fneur.2023.1303434 (PMC10800518; doi:10.3389/fneur.2023.1303434)
Supplement: Supplementary Table 1 — Demographics and clinical features of previously reported cases*. This table shows demographics and clinical features of previously reported cases of PD with MG in details, including their sex, age, age at onset of PD/MG, PD clinical features, MG clinical features, MG diagnosis, PD treatment and MG treatment. [file Table_1.docx]

Supplementary Material

# Supplementary Table

**Supplementary Table** Demographics and clinical features of previously reported cases.* This table shows demographics and clinical features of previously reported cases of PD with MG in details, including their sex, age, age at onset of PD/MG, PD clinical features, MG clinical features, MG diagnosis, PD treatment and MG treatment.

| Year  Reference | Sex | Age | AAO of  PD/MG | PD Clinical Features | MG Clinical Features | MG Diagnosis | PD Treatment | MG Treatment |
| --- | --- | --- | --- | --- | --- | --- | --- | --- |
| 1987(1) | M | 60 | 55/60, 1 week after the start of THP treatment | Tremor, hypomimia | Head drop, weakness of shoulder muscles, ptosis, diplopia, dysphagia | anti-AchR Ab (+)  ET (+)  RNS (+) | L-dopa | Pyd 180 mg/d for 2 weeks → thymectomy |
| 1991(2) | F | 77 | 74/77 | Tremor, axial rigidity, hypomimia | Fatigue, ptosis, diplopia, dysphagia, dysarthria | ET (+)  RNS (+) | Amantadine | Anticholinesterase drugs |
| 1993(3) | F | 62 | 54/61 | Bradykinesia, tremor, limb rigidity, postural instability, hypomimia | Fluctuating left eye ptosis, diplopia, slurred speech, dysphagia, generalized muscle weakness | anti-AchR Ab (+)  ET (+)  RNS (+) | Madopar 875 mg/d | Pyd 240–360 mg/d→ PDN 60 mg/d until improvement |
| 2003(4) | M | 81 | 76/81 | Resting Tremor, rigidity | Head drop | anti-AchR Ab (+)  ET (+) | L-dopa | Pyd, AZA |
| 2003(4) | M | 66 | 62/66 | Unilateral tremor, rigidity | Intermittent unilateral ptosis, lower limb weakness, diplopia, dysmasesia, dyspnea | anti-AchR Ab (+)  SFEMG (+) | L-dopa, pergolide, selegiline, amantadine | Pyd |
| 2003(4) | F | 71 | 71/68 | Rigidity, leg restlessness | Limb weakness, ptosis | anti-AchR Ab (+) | L-dopa | Pyd, AZA |
| 2003(4) | M | 61 | 61/data not shown | Rigidity, hypophonia | Bilateral ptosis | SFEMG (+) | L-dopa | Pyd |
| 2008(5) | F | 58 | 53/58 | Right hand resting tremor, rigidity, bradykinesia | Head drop | anti-AchR Ab (-)  anti-MuSK Ab (-)  ET (+)  RNS (-)  SFEMG (+) | Data not shown | Pyd 60 mg q.i.d., PDN 50 mg/d, AZA 125 mg/d → plasma exchange for 6 sessions → PDN 5 mg/d |
| 2009(6) | M | 84 | 80/84 | Resting tremor, rigidity, and bradykinesia | Head drop | anti-AchR Ab (-)  NT (+)  RNS (-)  SFEMG (+) | Data not shown | Pyd 60 mg q.i.d. |
| 2011(7) | M | 75 | 67/75 | Resting tremor, rigidity | Head drop | anti-AchR Ab (+)  RNS (+)  SFEMG (+) | L-dopa + benserazide (250 mg/d) | ivIg for 5 days, Pyd 180 mg/d for 3 months |
| 2014(8) | F | - | 68/75 | Right-hand resting tremor, stiffness, slowness | Weakness of the limbs and neck flexor muscles | anti-AchR Ab (+) | Carbidopa–levodopa | Pyd, AZA → ivIg 2 g/kg every 6 weeks for 8 years |
| 2014(8) | M | 77 | 76/72 | Resting tremor, stiffness, slowness | Ptosis, ophthalmoplegia | anti-AchR Ab (+) | Symptomatic therapy was not started then | Low-dosage Pyd |
| 2014(9) | M | 69 | 64/69 | Left hand rigidity | Head drop | anti-AchR Ab (+)  anti-MuSK Ab (-)  RNS (+) | Data not shown | Pyd 60 mg q.i.d., PDN 10 mg/d → PDN to 75 mg/d due to RA |
| 2016(10) | F | 67 | 57/66 | Data not shown | Bilateral ptosis, asthenia | anti-AchR Ab (-)  NT (+)  SFEMG (+) | Data not shown | Pyd 15–30 mg q.i.d. |
| 2016(10) | M | 64 | 60/63 | Data not shown | Bilateral ptosis, head drop | anti-AchR Ab (-)  NT (+)  RNS (+)  SFEMG (+) | Data not shown | Pyd 15–30 mg q.i.d. |
| 2016(10) | M | 72 | 67/70 | Data not shown | Ptosis, diplopia, asthenia | anti-AchR Ab (+)  NT (+)  SFEMG (+) | Data not shown | Pyd 15–30 mg q.i.d. |
| 2016(11) | M | 68 | 68/66 | Bradykinesia, speech disorder | Data not shown | Data not shown | Levodopa-benserazide  HCl 60 mg t.i.d. | Pyd 60 mg q.i.d. |
| 2016(12) | F | 90 | 81/90 | Bradykinesia, hypomimia, rigidity, resting tremor | Sudden severe dysphagia, dysarthria, profuse sialorrhea, worsening in visual acuity | anti-AchR Ab (-)  NT (+)  RNS (+)  SFEMG (+) | L-dopa | Pyd 60 mg t.i.d., PDN 30 mg/d, AZA 100 mg/d → (1 week later) ivIg 0.5 g/kg (25 g) for 5 days |
| 2016(13) | M | 73 | 53/data not shown | Bradykinesia, rigidity | Fluctuating ptosis, diplopia, dysarthria, dysphagia, general weakness | anti-AchR Ab (+)  NT (+)  RNS (+) | Data not shown | Pyd 120 mg/d |
| 2016(14) | F | 76 | 71/76 | Mild rigidity, akinesia | Head drop | anti-AchR Ab (+)  ET (+)  RNS (+) | L-dopa 300 mg/d (with 30 mg/d carbidopa) | Pyd 60 mg/d, PDN 30 mg/d |
| 2017(15) | M | 75 | 75/75 | Hypomimia, hypophonia, neck rigidity, bradykinesia, shuffling gait | Head drop, nasal dysarthria, dysphagia, diplopia | anti-AchR Ab (+)  anti-MuSK Ab (-) | L-dopa | Pyd, ivIg, custom head brace |
| 2018(16) | M | 82 | 76/data not shown | Freezing of gait | Progressive dysphagia | anti-AchR Ab (+)  ET (+)  RNS (-) | Data not shown | Pyd 240 mg/d → + PDN 20 mg/d+ AZA  2.5 mg/kg/d followed by ivIg for 5 days |
| 2018(16) | M | 95 | 90/94 | Data not shown | Progressive dysphagia | anti-AchR Ab (+)  RNS (-) | Data not shown | Pyd 330 mg/d, PDN 20 mg/d |
| 2018(16) | M | 83 | 78/data not shown | Mobility-impaired, sialorrhea | Progressive dysphagia | anti-AchR Ab (+)  ET (+)  RNS (-) | Data not shown | Pyd 120 mg/d, PDN 10 mg/d, AZA |
| 2018(16) | M | 81 | 74/data not shown | Mobility-impaired | Progressive dysphagia | anti-AchR Ab (+)  ET (+)  RNS (-) | Data not shown | Pyd 210 mg/d, ivIg 0.4g/kg for 5 days → PDN 20 mg/d, AZA |
| 2019(17) | M | 73 | 65/73 | Resting tremor, bradykinesia, rigidity | Dysphagia, fatigue, diplopia | anti-AchR Ab (+)  NT (+)  RNS (+) | L-dopa, carbidopa, safinamide | Pyd, PDN, AZA |
| 2019(18) | M | 60 | 49/60 | Bradykinesia, rigidity, hypomimia, vertical down-gaze limitation of the left eye | Right palpebral ptosis, vertical diplopia | anti-AchR Ab (+)  NT (+)  RNS (-) | Levodopa/Carbidopa/Entacapone 150/37.5/200 mg 6 ×/d, Ropinirole 16 mg q.d., Rasagiline 1 mg q.d., Madopar HBS (Levodopa/Benserazide) 100/25 mg q.d. | Neostigmine 60 mg b.i.d. |
| 2019(18) | F | 69 | 64/68 | Right upper-limb kinetic disability | Palpebral ptosis, dysarthria, dysphagia | anti-AchR Ab (-)  NT (+)  RNS (-) | Levodopa/Carbidopa/Entacapone 100/25/200 mg t.i.d., pramipexole 1.05 + 0.26 mg, Rasagiline 1 mg q.d. → Levodopa/Carbidopa/Entacapone 500 mg/d 5×/d, the dose of the Das was decreased | Neostigmine 60 mg t.i.d. |
| 2019(18) | M | 64 | 52/55 | Global bradykinesia, axial rigidity, rest tremor | Incomplete palpebral ptosis, horizontal diplopia | anti-AchR Ab (-)  NT (+)  RNS (-) | Levodopa/Carbidopa/Entacapone 150/37.5/200 mg t.i.d., Rasagiline 1 mg q.d., Ropinirole 8 mg/d | Neostigmine 60 mg q.i.d. |
| 2021(19) | M | 73 | Data not shown/71.5 | asymmetrical limbs rigidity, and bradykinesia | gradual progressive dysphagia, generalized weakness, fatigue, and slowness of movement and gait, diplopia | anti-AchR Ab (-)  anti-MuSK Ab (+)  RNS (-) | levodopa/carbidopa | ivIg 0.4 g/kg/day→five sessions of plasmapheresis and intravenous high dose methylprednisone, high-dose prednisone and  Pyd 60 mg q.i.d., azathioprine→rituximab |
| 2022(20) | M | - | 83/84 | Bradykinesia and tremor | Ptosis, diplopia, dysarthria, dysphonia and dysphagia | anti-AchR Ab (+) | L-dopa | AChEI, steroids |
| 2022(20) | M | - | 66/68 | Bradykinesia and tremor | Ptosis and diplopia | anti-AchR Ab (-) | None | None |
| 2022(20) | M | - | 62/64 | Bradykinesia and tremor | Ptosis and diplopia | anti-AchR Ab (+) | L-dopa | AChEI, steroids |
| 2022(20) | M | - | 50/75 | Bradykinesia, tremor and rigidity | Ptosis, diplopia, dysarthria, dysphonia and dysphagia | anti-AchR Ab (-) | L-dopa | AChEI |
| 2022(20) | M | - | 59/62 | Bradykinesia, tremor and rigidity | Ptosis, diplopia, and dropped head | anti-AchR Ab (+) | L-dopa | AChEI, steroids |
| 2022(20) | M | - | 66/69 | Bradykinesia, tremor and rigidity | Ptosis and diplopia | anti-AchR Ab (-) | L-dopa | AChEI, steroids |
| 2022(20) | M | - | 59/60 | Bradykinesia, tremor and rigidity | Ptosis and diplopia | anti-AchR Ab (-) | L-dopa | AChEI, steroids |
| 2022(20) | M | - | 65/67 | Bradykinesia and tremor | Ptosis, diplopia and dropped head | anti-AchR Ab (+) | L-dopa | AChEI, steroids, immunosuppressant |
| 2022(20) | M | - | 83/72 | Bradykinesia and tremor | Ptosis and diplopia | anti-AchR Ab (-) | L-dopa | AChEI, steroids |
| 2022(20) | F | - | 84/84 | Bradykinesia and tremor | Ptosis, diplopia, dysarthria, dysphonia and dysphagia | anti-AchR Ab (+) | L-dopa | AChEI, steroids |
| 2022(20) | M | - | 73/75 | Bradykinesia and tremor | Ptosis and diplopia | anti-AchR Ab (-) | L-dopa | AChEI, steroids |
| 2022(20) | M | - | 76/75 | Bradykinesia and tremor | Ptosis, diplopia and dropped head | anti-AchR Ab (-) | L-dopa | AChEI |

*We summarized demographics and clinical features of 42 cases and a case series(21) was not included in the table as it didn’t provide much detailed information. The retrospective observational study of 12 cases from a single center were listed(20) at the last few rows of the table and it didn’t provide some information, such as the age, more specific clinical manifestation of PD and the results of edrophonium/neostigmine test or electromyography of each patient.

AAO, age at onset, PD, Parkinson’s disease, MG, myasthenia gravis

M, male, F, female, THP, trihexyphenidyl, AchR, acetylcholine receptor, MuSK, muscle-specific tyrosine kinase, Ab, antibody

ET, edrophonium test, NT, neostigmine test, RNS, repetitive nerve stimulation, SFEMG, single fiber electromyogram

L-dopa, levodopa, Pyd, pyridostigmine, PND, prednisone, AZA, azathioprine, i.v., intravenous, ivIg, intravenous immunoglobulin, DAs, dopamine agonists, AChEI, acetylcholinesterase inhibitors

RA, rheumatoid arthritis

# References

1. Ueno S, Takahashi M, Kajiyama K, Okahisa N, Hazama T, Yorifuji S, et al. Parkinson's Disease and Myasthenia Gravis: Adverse Effect of Trihexyphenidyl on Neuromuscular Transmission. Neurology (1987) 37(5):832-3. Epub 1987/05/01. doi: 10.1212/wnl.37.5.832.

2. Tasic Z, Stefanovic P, Apostoloski S. An Unusual Association of Myasthenia Gravis and Parkinsonism in a Female Patient with Tuberculous Lymphadenitis. Srp Arh Celok Lek (1991) 119(3-4):103-6. Epub 1991/03/01.

3. Kao KP, Kwan SY, Lin KP, Chang YC. Coexistence of Parkinson's Disease and Myasthenia Gravis: A Case Report. Clin Neurol Neurosurg (1993) 95(2):137-9. Epub 1993/06/01. doi: 10.1016/0303-8467(93)90008-5.

4. Levin N, Karussis D, Abramsky O. Parkinson's Disease Associated with Myasthenia Gravis. A Report of 4 Cases. J Neurol (2003) 250(6):766-7. Epub 2003/07/17. doi: 10.1007/s00415-003-1088-y.

5. Fasano A, Evoli A, Piano C, Tonali PA, Bentivoglio AR. Myasthenia Gravis: An Unrecognized Cause of Head Drop in Parkinson's Disease. Parkinsonism Relat Disord (2008) 14(2):164-5. Epub 2007/04/27. doi: 10.1016/j.parkreldis.2007.02.009.

6. Unal-Cevik I, Temucin CM. Head Drop in an Elder Parkinson's Disease after Development of Myasthenia Gravis. Mov Disord (2009) 24(13):2025-6. Epub 2009/08/01. doi: 10.1002/mds.22704.

7. Uludag IF, Korucuk M, Sener U, Zorlu Y. Myasthenia Gravis as a Cause of Head Drop in Parkinson Disease. Neurologist (2011) 17(3):144-6. Epub 2011/05/03. doi: 10.1097/NRL.0b013e3182173356.

8. Neuman LA, Cheema FZ. Two Cases of Parkinson Disease and Concurrent Myasthenia Gravis, Generalized and Ocular. Neurohospitalist (2014) 4(3):117-8. Epub 2014/07/02. doi: 10.1177/1941874414522498.

9. Zis P, Argiriadou V, Temperikidis PP, Zikou L, Tzartos SJ, Tavernarakis A. Parkinson's Disease Associated with Myasthenia Gravis and Rheumatoid Arthritis. Neurol Sci (2014) 35(5):797-9. Epub 2014/02/07. doi: 10.1007/s10072-014-1660-5.

10. Sciacca G, Nicoletti A, Mostile G, Dibilio V, Raciti L, Luca A, et al. Is It Just a Coincidence? Three New Cases of Myasthenia Gravis Associated with Parkinson's Disease. Parkinsonism Relat Disord (2016) 28:166-8. Epub 2016/04/16. doi: 10.1016/j.parkreldis.2016.04.005.

11. Ozer AB, Catak T, Ozdemir M, Kilinc M. Anesthesia Management in the Coexistence of Myasthenia Gravis and Parkinsonism. J Clin Anesth (2016) 34:350-1. Epub 2016/10/01. doi: 10.1016/j.jclinane.2016.05.033.

12. Tung-Chen Y, Bataller L, Sevilla T, Lopez-Aldeguer J. Co-Occurrence of Myasthenia Gravis with Parkinson's Disease: A Not to Be Missed Diagnosis. Geriatr Gerontol Int (2016) 16(4):528-30. Epub 2016/04/03. doi: 10.1111/ggi.12558.

13. Beyrouti R, Courtois S. Parkinson Disease Associated with Myasthenia Gravis: A Case Report and Literature Review. Journal of Neurological Disorders (2016) 4(4). doi: 10.4172/2329-6895.1000283.

14. Aiba Y, Iwakawa M, Sakakibara R, Tsuyusaki Y, Tateno F, Kishi M, et al. Myasthenia Gravis Manifesting as Head Drop in an Elderly Adult with Parkinson's Disease. J Am Geriatr Soc (2016) 64(10):e120-e2. Epub 2016/10/21. doi: 10.1111/jgs.14359.

15. Hogg EJ, Lewis RA, Bannykh S, Tagliati M. Head Drop in Parkinson's Disease Complicated by Myasthenia Gravis and Myopathy. J Neurol Sci (2017) 376:216-8. Epub 2017/04/23. doi: 10.1016/j.jns.2017.03.026.

16. Urban PP, Stammel O. Myasthenia Gravis Should Be Considered in Cases of Parkinson's Disease and Progressive Dysphagia. Nervenarzt (2018) 89(4):443-5. Epub 2017/07/13. doi: 10.1007/s00115-017-0378-z.

17. Marano M, Lanzone J, di Biase L, Pepe A, Di Santo A, Di Lazzaro V. A Rare Cause of Axial Worsening in Parkinson's Disease: A Case of Myasthenic Pseudo-Parkinsonism. Clin Neurol Neurosurg (2019) 179:1-3. Epub 2019/02/18. doi: 10.1016/j.clineuro.2019.02.009.

18. Odajiu I, Davidescu EI, Mitu C, Popescu BO. Patients with Parkinson's Disease and Myasthenia Gravis-a Report of Three New Cases and Review of the Literature. Medicina (Kaunas) (2019) 56(1). Epub 2019/12/28. doi: 10.3390/medicina56010005.

19. Albassam MS, Thabet SA, Hmoud M, Makkawi S. Anti-Muscle Specific Kinase (Anti-Musk) Positive Myasthenia Gravis Overlapping with Parkinson's Disease: A Challenging Diagnosis. Cureus (2021) 13(5):e14839. Epub 2021/06/10. doi: 10.7759/cureus.14839.

20. Iori E, Mazzoli M, Ariatti A, Salviato T, Rispoli V, Valzania F, et al. Myasthenia Gravis Crossing Parkinson's Disease: A 20 Year Study from Single Italian Center. Int J Neurosci (2022):1-7. Epub 2022/08/03. doi: 10.1080/00207454.2022.2107517.

21. Alshaikh JT, Mills K. Coincident Parkinsonism and Myasthenia Gravis: A Case Series. Parkinsonism Relat Disord (2021) 89:4-5. Epub 2021/07/02. doi: 10.1016/j.parkreldis.2021.06.016.
